# Impact of a long-term mentorship program for childbirth providers on quality of care: A cluster randomized controlled trial

**DOI:** 10.1371/journal.pgph.0006073

**Published:** 2026-09-02

**Authors:** Lujia Zhang, Rajet Vatsa, Wei Chang, Sharon Akinyi, Sarah Little, Catherine Gakii, John Mungai, Cynthia Kahumbura, Sathyanath Rajasekharan, Anneka Wickramanayake, Margaret McConnell, Jessica Cohen

**Affiliations:** 1 Department of Global Health and Population, Harvard T. H. Chan School of Public Health, Boston, Massachusetts, United States of America; 2 Harvard/MIT MD-PhD Program, Harvard Medical School, Boston, Massachusetts, United States of America; 3 Africa Chief Economist Office, World Bank, Washington, District of Columbia, United States of America; 4 Jacaranda Health, Nairobi, Nairobi County, Kenya; 5 Innovations for Poverty Action, Nairobi, Nairobi County, Kenya; University of California San Francisco, UNITED STATES OF AMERICA

## Abstract

Maternal and neonatal mortality rates remain high in sub-Saharan Africa despite increased facility-based childbirth, reflecting persistent gaps in quality of care. We evaluated the impact of MENTORS, a facility based nurse mentorship and training program, on provider knowledge and childbirth care quality. In an unblinded parallel-arm cluster randomized controlled trial conducted between November 2021, 40 health facilities in Kenya were randomized to receive continuing medical education lectures, simulation-based drills, and in-facility mentorship to strengthen obstetric and newborn care. Baseline data included facility assessments and provider surveys; endline data included provider surveys, a standardized neonatal resuscitation simulation, and childbirth observations. Primary outcomes included provider knowledge, quality of routine maternity care, and quality of neonatal resuscitation management. All outcomes were assessed at endline. The trial was registered at ClinicalTrials.gov (NCT05110521) and the AEA RCT Registry (0008449). At baseline (November-December 2021), 357 providers were interviewed; at endline (May-August 2022), 376 providers were interviewed and 4,377 childbirths were observed. Routine maternity care quality was low across facilities, with providers performing 58.6% of essential actions in intervention facilities and 57.0% in control facilities at endline. There was no significant difference between the intervention and control facilities in provider knowledge (unadjusted domain effect 0.006, 95% CI: -0.038, 0.051) nor the quality of routine maternity care (0.018, 95% CI: -0.029, 0.065). However, providers in intervention-exposed facilities demonstrated improved quality of neonatal resuscitation in simulation-based assessments (0.094, 95% CI: 0.000, 0.188), with suggestive evidence of improved performance in standardized childbirth observations as well (0.041, 95% CI: -0.001, 0.083). Routine maternity care quality was suboptimal across both intervention and control facilities. While the facility-based mentorship program did not significantly improve provider knowledge or the quality of routine childbirth care, it showed evidence of improvements in neonatal resuscitation, although these findings were not robust across all sensitivity analyses.

## Introduction

Globally, maternal mortality rates reveal stark disparities: 10 per 100,000 live births in high-income countries compared to 346 per 100,000 in low-income countries, with sub-Saharan Africa accounting for 70% of global maternal deaths [1]. Like much of sub-Saharan Africa, Kenya continues to face high maternal mortality, estimated at 379 maternal deaths per 100,000 live births [1]. These figures reflect persistent challenges including inadequate access to emergency obstetric care and problems with the quality of maternal health services [2,3].

While significant strides have been made over the past few decades in increasing access to routine maternal and newborn care and the rate of facility-based childbirth in sub-Saharan Africa, [4] several studies show that higher institutional childbirth rates have not consistently translated into better outcomes [5,6]. Women continue to experience both “too little, too late”, care that is delayed, unavailable, or delivered with inadequate resources, and “too much, too soon”, the overuse of non-evidence-based or unnecessary interventions [7]. In Kenya, quality of maternity care is highly variable and often lacking, characterized by unnecessary interventions such as C-sections, disrespectful treatment, and non-adherence to routine clinical guidelines [8,9].

Recent efforts to improve maternal and newborn health have largely focused on training healthcare providers. Global initiatives have emphasized “skills and drills”, simulation exercises, and short courses typically 1–5 days in length, designed to strengthen clinical performance and emergency response [10,11]. Evaluations across low-and-middle income countries have documented improved adherence to clinical protocols, and greater provider knowledge, but effects on maternal and newborn health outcomes have been mixed [10,12,13]. A potential reason for the modest effect of these programs on health outcomes is the limited nature of the training. Without repeated practice, reinforcement, or sustained supervision, providers may struggle to integrate newly learned practices into routine clinical care [14].

Mentorship programs have been proposed as an alternative. These multi-month longitudinal programs typically involve more experienced healthcare providers working with less experienced mentees to improve their performance, support their professional development, and enable skills transfers [15]. Evidence to date suggests that mentorship programs can improve knowledge and competency, particularly in simulated settings [16–18]. However, cluster-randomized and stepped-wedge trials of training and coaching interventions have shown that improvements in knowledge and adherence to clinical practices do not consistently translate into better teamwork, clinical performance, or maternal and neonatal outcomes [19–21]. Further evidence is needed to determine whether these improvements translate into improved quality of care and health outcomes. Thus, there is a need to rigorously evaluate the impact of clinical training and mentorship programs on quality of maternity care, especially when scaled up in sub-Saharan African settings like Kenya [10,16].

In this paper, we conduct a randomized evaluation of MENTORS, a nurse mentorship and emergency obstetric and newborn care training program developed by Jacaranda Health, a maternal and child health organization in Kenya. Unlike most training programs that rely on short-term or one-off sessions, [10] MENTORS is designed to provide longitudinal, facility-embedded support for maternity care. We use direct childbirth observations and provider surveys from facilities across Kenya to measure quality of childbirth care, maternal and neonatal health outcomes, and provider knowledge.

## Methods

### Ethics statement

This study was licensed by the National Commission for Science, Technology, and Innovation in Kenya (NACOSTI/P/21/13369) and approved by the institutional review board of the Harvard T.H. Chan School of Public Health (IRB21–1013) and the ethics and scientific review committee of Amref Health Africa (P1047/2021). The trial was implemented in the 40 study facilities following approval of the study protocol by respective facility in-charges and county health directors. Following the study endline, control arm facilities also began receiving the intervention package.

All data were collected by enumerators who received extensive training in research ethics, proper conduct in maternity wards, and study procedures by the local implementation partner. Written, electronic, or thumbprint-based informed consent was obtained from all facility in-charges, health workers, and patients involved in the study. Only women who were stable and able to focus on the consent process were approached for participation. An emphasis on voluntary participation, participant confidentiality, and freedom to withdraw consent at any time was upheld throughout. Women under 18 years of age, but at least 15 years of age, were allowed to consent and participate in the study on their own volition, given pregnant individuals are considered mature minors in Kenya. To prevent undue harm to any patient, psychological supports and abridged protocols were offered in cases of stillbirth, miscarriage, or infant loss at the time of childbirth.

### Study design

We conducted an unblinded parallel arm cluster randomized controlled trial (RCT) between November 2021 and August 2022 in 40 public and faith-based health facilities across eight counties in Kenya (Fig A in S1 Appendix). The intervention, designed and implemented by Jacaranda Health, contained two parts: i) a nurse mentorship and emergency obstetric and newborn care training program (“MENTORS”) and ii) a digital health platform supporting pregnant and postpartum women through informational messages, appointment reminders, and a clinical helpdesk (“PROMPTS”). In this manuscript, we restrict our focus to the MENTORS program. Previous research from our team separately analyzed the impact of the PROMPTS program, finding the platform improved mothers’ antenatal and postpartum knowledge, preparedness, and health behaviors, but did not affect care seeking behavior for danger signs.[22]

Facilities selected for inclusion in the study were either government-owned or faith-based, had an average of 50–400 normal vaginal births per month (based on records from the Kenya Health Management Information System), and were in counties in which Jacaranda Health had a plan to expand. To ensure no overlap with concurrent interventions, eligible facilities were also required to have no ongoing mobile health or quality improvement research initiative. The birth volume criterion was set to enable sufficient statistical power for outcomes derived from standardized childbirth observations and because the MENTORS program primarily targets facilities of this size, namely mid- to high-volume facilities.

To ensure sufficient distance between study facilities to minimize the risk of spillovers, candidate facility locations were reviewed by the study team and in the case where two facilities were near each other (i.e., in the same sub-county), the lower-level or lower-volume facility was excluded. Block 1:1 randomization was done at the facility level and stratified based on tertile of normal vaginal birth volume. Following randomization, the minimum distance between facilities in different study arms was approximately 10 kilometers.

### Intervention

MENTORS is a facility-based provider-facing program aimed at increasing and sustaining skills in basic emergency obstetric and newborn care, which includes the timely recognition and management of common complications during childbirth and the immediate postpartum period. As of 2024, MENTORS had trained over 10,000 nurses in basic and emergency obstetric and newborn care across 400 + health facilities. To enable low-cost scale up, the program uses a “training of trainers” model (Fig B in S1 Appendix). Lead mentors underwent a structured training program conducted by Jacaranda Health, consisting of six weeks of full-time training that included both clinical content in basic and emergency obstetric and newborn care and supervised shadowing with experienced mentors. Lead mentors were assessed during the training period on clinical knowledge and skills, including performance on standardized knowledge assessments and neonatal resuscitation examinations, prior to deployment. Lead mentors helped facilitate mentorship activities within their respective counties and trained other providers to become in-facility mentors. In-facility mentors were selected from existing maternity staff in participating facilities, including nurse midwives, clinical officers, and medical officers, based on clinical experience and recommendations from county health leadership. These in-facility mentors received one week of full-time training with knowledge-based pretests and posttests and skill-based simulations. They subsequently led longitudinal training and mentorship for facility-based peers, including continuing medical education (CME) lectures, simulation-based drills, and childbirth debriefs.

Each participating facility is expected to hold a minimum of two CME lectures and one simulation-based drill every month offering hands-on opportunity to bridge classroom learning with clinical practice. The CME lectures consist of onsite and virtual sessions approximately 45 minutes in length that cover a wide range of topics including normal labor and childbirth, postpartum hemorrhage, neonatal resuscitation, shoulder dystocia, respectful maternity care, hypertensive disorders of pregnancy, complications of abortion, and infection prevention. Meanwhile, the drills consist of 1.5–2-hour long simulation-based trainings covering topics such as normal childbirth, shoulder dystocia, eclampsia, breech birth, and assisted vaginal birth. Mentors select topics based on their identification of facility needs and training equipment availability.

### Data

Data were collected by trained enumerators at multiple points throughout the study period (Fig C in S1 Appendix). Baseline data collection, which occurred from November 5^th^ 2021 to December 7^th^ 2021, consisted of a facility assessment and provider survey. Endline data collection occurred from May 2^nd^ 2022 to August 3^rd^ 2022, and consisted of a provider survey, neonatal resuscitation simulation, and childbirth observations. Endline data collection was split into two waves based on geographic location, with 20 facilities per wave, and lasted for one to two months in each facility depending on the number of childbirths observed. 90 enumerators conducted childbirth observations across the two waves, with three to four enumerators collecting data per facility. Enumerators observed births at least four days per week, including on weekends and overnight.

**Facility Assessment:** Enumerators conducted a baseline facility assessment with facility staff collecting information on facilities’ infrastructure, staffing, available procedures, medical supplies and equipment, referral management, treatment protocols, and patient-borne costs associated with maternity services, using tools adapted from prior studies [23].

**Provider Survey:** Maternity ward providers were eligible for the provider baseline survey if they had no plans to move to a different facility within the following 6 months. Providers were interviewed about their clinical education, experience with different maternity services, knowledge of recommended maternal and newborn health care practices, recent in-service training in obstetrics, and workplace motivation. The endline provider surveys included the same elements as the baseline survey and added a standardized simulation experience, previously validated in Tanzania, to assess the newborn resuscitation skills of birth attendants [24].

**Childbirth Observations:** Childbirth observations began approximately 5 months after the intervention began. Enumerators – who themselves were required to have pursued or graduated with an accredited medical, nursing, or midwifery degree and to have experience in maternal and newborn health – received training on the observation tools under the supervision of OBGYNs and midwives. They were trained to minimize disruption to care processes and were instructed to intervene only in the case of an avertable life-threatening emergency. The childbirth observations involved a detailed checklist of procedures in routine and emergency obstetric and newborn care, adapted from a checklist previously used in Kenya [25]. Emergency obstetric care was recorded for four specific types of complications: neonatal resuscitation, postpartum hemorrhage, shoulder dystocia, and severe pre-eclampsia/eclampsia. Maternal data (e.g., age, marital status, parity, gravidity) and newborn details (e.g., birthweight, sex) were obtained from the Ministry of Health maternity register following review of patient files after discharge.

### Outcomes

The outcomes for this study were pre-registered on the United States Clinical Trials Registry (NCT05110521; registered 10/25/2021) and American Economic Association RCT Registry (AEARCTR-0008449; registered 10/28/2021). Primary and secondary outcomes were organized within pre-specified domains to account for multiple comparisons and reduce the risk of false positives.

Pre-registered primary outcomes for the MENTORS component of the study included provider knowledge (of obstetric and newborn care), quality of routine maternity care, and quality of neonatal resuscitation management. Provider knowledge was assessed in the endline survey as the proportion of correct responses to ten knowledge questions on normal labor, infection prevention and control, pre-eclampsia/eclampsia, postpartum hemorrhage, newborn care, and shoulder dystocia. Quality of routine maternity care was measured from childbirth observations as the share of essential actions that providers performed, based on an equally weighted 20-item index published by Tripathi et al [26]. The Tripathi index has been previously validated in Kenya, with higher scores denoting better quality of care. In exploratory analyses, we disaggregated care quality across the four stages of the Tripathi index (initial client assessment and examination, first stage of labor, second and third stage of labor, and immediate newborn and postpartum care). Finally, quality of neonatal resuscitation management was measured using a structured, scenario-based simulation based on the Helping Babies Breathe curriculum [27]. Providers were presented with a standardized clinical scenario of a non-breathing newborn following a complicated delivery and were asked to demonstrate resuscitation using a mannequin and standard equipment. Enumerators guided the simulation by providing clinical information (e.g., heart rate) in response to provider assessments. Performance was evaluated using a standardized checklist of essential resuscitation steps, and providers were assessed on the fraction of equally weighted steps performed correctly. We also assessed, as an exploratory outcome, whether providers achieved a perfect score on the neonatal resuscitation simulation as all elements of the simulation need to occur for a resuscitation to be successful in practice.

Pre-registered secondary outcomes included an index of provider motivation and satisfaction, measured via a scale validated in Kenya by Mbindyo et al. (2009); [28] an index of quality of management of maternal and neonatal complications (i.e., neonatal resuscitation, postpartum hemorrhage, shoulder dystocia, severe pre-eclampsia/eclampsia); an index of quality of infection prevention; an index of quality of partograph completion; an index of provider treatment of patients during labor and childbirth, assessed using an interpersonal abuse and unsupportive birth environment measure developed by Berger et al. (2022); [29] a composite index of severe maternal and neonatal morbidity (i.e., postpartum hemorrhage, severe pre-eclampsia, eclampsia, maternal sepsis, neonatal sepsis or systemic infection, uterine rupture, neonatal asphyxia, maternal mortality, and perinatal mortality); and timeliness of care delivery (e.g., between facility arrival and first contact with a provider). In exploratory analyses, we evaluated process- and outcome-based measures for individual complications observed by enumerators with sufficient frequency during childbirth observations, namely neonatal resuscitation and postpartum hemorrhage. The outcomes included quality of neonatal resuscitation management, successful neonatal resuscitation, quality of postpartum hemorrhage management, and stabilization (i.e., controlling or ending) of postpartum bleeding. A detailed description of each primary and secondary outcome, including specific index components, is provided in Table A in S1 Appendix.

### Power

Based on prior work by members of our study team, we assumed that the mean quality of routine maternity care in the control group would be 58%, with a standard deviation of 13%. With an expected 100 fully observed childbirths per cluster, 20 clusters per study arm, intra-cluster correlation of 0.35, significance level of 0.05, and power of 80%, we calculated a minimum detectable effect size of 6.88 percentage points.

### Statistical analysis

We first descriptively assessed baseline characteristics of facilities, providers, and patients whose births were observed in the intervention and control facilities. We then examined, at endline, provider attendance at in-service trainings within the preceding six months. To assess the impact of the MENTORS program, we estimated the intention-to-treat effect on provider-level and individual childbirth observation-level outcomes at endline using linear models. Outcomes were regressed on a binary variable indicating whether the facility was in the intervention group, the baseline facility birth volume tertile (stratification variable), and an indicator for whether the endline data were collected in wave 1 or 2. We did not collect and therefore do not adjust for measures of each outcome at baseline. Accordingly, our findings should be interpreted as endline differences rather than change-from-baseline effects. For childbirth observation-level outcomes, models were estimated on the sample of fully observed childbirths. Due to the facility-level randomization, heteroskedasticity robust standard errors were used and clustered at the facility level. For select exploratory outcomes, we generated figures to visualize qualitative trends. Analyses and data visualization were done in Stata version 17 and R version 4.2.1.

### Sensitivity analysis

We conducted three sensitivity analyses. First, to improve precision and account for potential facility- and county-level differences, we estimated adjusted models that included controls for facility and county. Second, although primary and secondary outcomes were organized within pre-specified domains to account for multiple comparisons and reduce the risk of false positives, we additionally assessed robustness to multiple hypothesis testing by applying Benjamini-Hochberg adjustments to the P-values for the pre-registered outcomes. Third, during baseline data collection, we identified three facilities in the intervention arm that were concurrently participating in other research studies aimed at improving care for specific obstetric emergencies, and we repeated analyses excluding these facilities.

## Results

Among the 40 study facilities, five were Level 3 health centers, 32 were Level 4 hospitals, and three were Level 5 county referral hospitals. A total of 360 providers were recruited at baseline (~60% of all maternity care providers in study facilities), and 357 were eligible and consented to be interviewed. Meanwhile, 376 providers were recruited at endline, all of whom consented to be interviewed; 242 (68%) of these providers were also interviewed at baseline.

Facility and provider characteristics were well-balanced between the intervention and control groups at baseline (Table 1). On average, facilities had 15 providers working in the maternity department and 23 beds for maternity care. Nearly all facilities (98%) offered around-the-clock obstetric and neonatal care. On average, facilities were equipped with 76% of the essential equipment for maternity care and 90% of essential drug categories. 68% of facilities had a functional blood bank. The number of providers interviewed at each facility ranged from 5-18. Most providers (78%) were female, with an average age of 25. Providers on average had worked at the study facility for 4.3 years, with 5.4 years of experience working in maternity care. 10% of providers had at least a bachelor’s degree.

**Table 1:** Baseline characteristics of study facilities and providers.

Baseline Characteristic	Control	Intervention
**Facility-Level**	**N = 20**	**N = 20**
# of providers in facility, mean (SD)	58.65 (37. 61)	57.10 (29.73)
# of providers in maternity care, mean (SD)	13.35 (4.49)	17.20 (10.03)
# of rooms in maternity unit, mean (SD)	5.25 (3.49)	5.35 (2.43)
# of beds in maternity unit, mean (SD)	23.50 (20.30)	23.30 (14.39)
Availability of 24/7 obstetric and neonatal care	20 (100%)	19 (95%)
Fraction of equipment/supplies available and working (out of 25)^1^, mean (SD)	0.74 (0.11)	0.77 (0.08)
Presence of a visitor waiting room	8 (40%)	11 (55%)
Availability of a sink with running water and soap or hand sanitizer	18 (90%)	20 (100%)
Patient referrals routinely made to other facilities	13 (65%)	15 (75%)
Availability of at least one functional mode of transportation for emergency referrals	7 (35%)	11 (55%)
Fraction of emergency obstetric care signal functions performed within the past three months (out of 9)^2^, mean (SD)	0.71 (0.21)	0.77 (0.17)
Availability of a pharmacy or drugstore 24 hours a day for staff	17 (85%)	12 (60%)
Fraction of essential drug categories available (out of 11)^3^, mean (SD)	0.90 (0.11)	0.90 (0.11)
Availability of a functional blood bank^4^	14 (70%)	13 (65%)
**Provider-Level**	**N = 177**	**N = 180**
Female	140 (79%)	137 (76%)
Age in years, mean (SD)	34.1 (8.27)	36.2 (9.12)
Bachelor’s or higher education	14 (8%)	21 (12%)
Years worked at current facility, mean (SD)	4.10 (5.16)	4.52 (5.95)
Years worked in maternity care across career, mean (SD)	4.96 (4.30)	5.83 (4.90)
# of types of maternity services provided in last three months (out of 7)^5^, mean (SD)	6.18 (1 ∙ 57)	5.82 (1.58)
Knowledge index (out of 1)^6^	0.50 (0.13)	0.53 (0.12)
# of in-service trainings related to obstetric care completed (out of 8)^7^, mean (SD)	5.08 (2.48)	5.26 (2.46)
Motivation index (out of 1)^8^	0.79 (0.09)	0.80 (0.10)

Notes:

1. Equipment/supplies assessed for include: sphygmomanometer, stethoscope, fetoscope, fetal Doppler, cardiotocography machine, wall clock or watch, infant or child weighing scale, neonatal Ambu bag and mask, incubator, autoclave equipment, cord clamps, mucus suction apparatus, delivery table, vacuum extractor, manual vacuum aspiration kit, thermometer, examination lamp/torch, sterile latex gloves (wrist/elbow length), disinfectant, partograph, sterile syringes, sutures, stainless steel bowls, kidney dishes, and delivery kits.

2. Nine signal functions assessed for include: administration of parenteral antibiotics, administration of uterotonic drugs, administration of parenteral anticonvulsants for pre-eclampsia and eclampsia, manual removal of placenta, removal of retained products of conception, assisted vaginal delivery, newborn resuscitation, blood transfusion, and surgery.

3. Essential drug categories assessed include: antibiotics, anticonvulsants, hydralazine, uterotonics, intravenous (IV) fluids, antiretrovirals, immunizations, Vitamin K injections, antihypertensives, chlorhexidine, and corticosteroids.

4. Facilities were asked this question directly without specifying the definition of a functional blood bank. Either the Head Laboratory Technician or other staff member with knowledge of such matters were approached to answer.

5. Types of maternity services assessed for include: administration of parenteral antibiotics, administration of anticonvulsants and uterotonics, manual removal of placenta, newborn resuscitation, assisted vaginal delivery, cesarean section, and blood transfusion.

6. Provider knowledge, scored from 0-1, was calculated as the fraction of correct responses to questions on the following 10 topics: causes of postpartum hemorrhage, diagnosis of pre-eclampsia, infection prevention, labor monitoring, newborn care, postpartum hemorrhage prevention, pre-eclampsia symptoms, shoulder dystocia complications, shoulder dystocia management, and vaginal examination infection control.

7. Providers were asked how many in-service trainings they had ever attended across the following eight obstetric topics: routine labor care, postpartum hemorrhage, neonatal resuscitation, shoulder dystocia, hypertensive disorders of pregnancy (including pre-eclampsia/eclampsia), respectful maternity care, infection prevention and control, and breech delivery.

8. Provider motivation, scored from 0-1, was calculated based on 11 Likert scale questions asking about job motivation and satisfaction. Responses for each question (on a 1–5 scale, with 1 denoting “strongly disagree” and 5 denoting “strongly agree”) were summed and divided by 55. A higher score corresponds to higher self-rated motivation.

**Fig 1:**
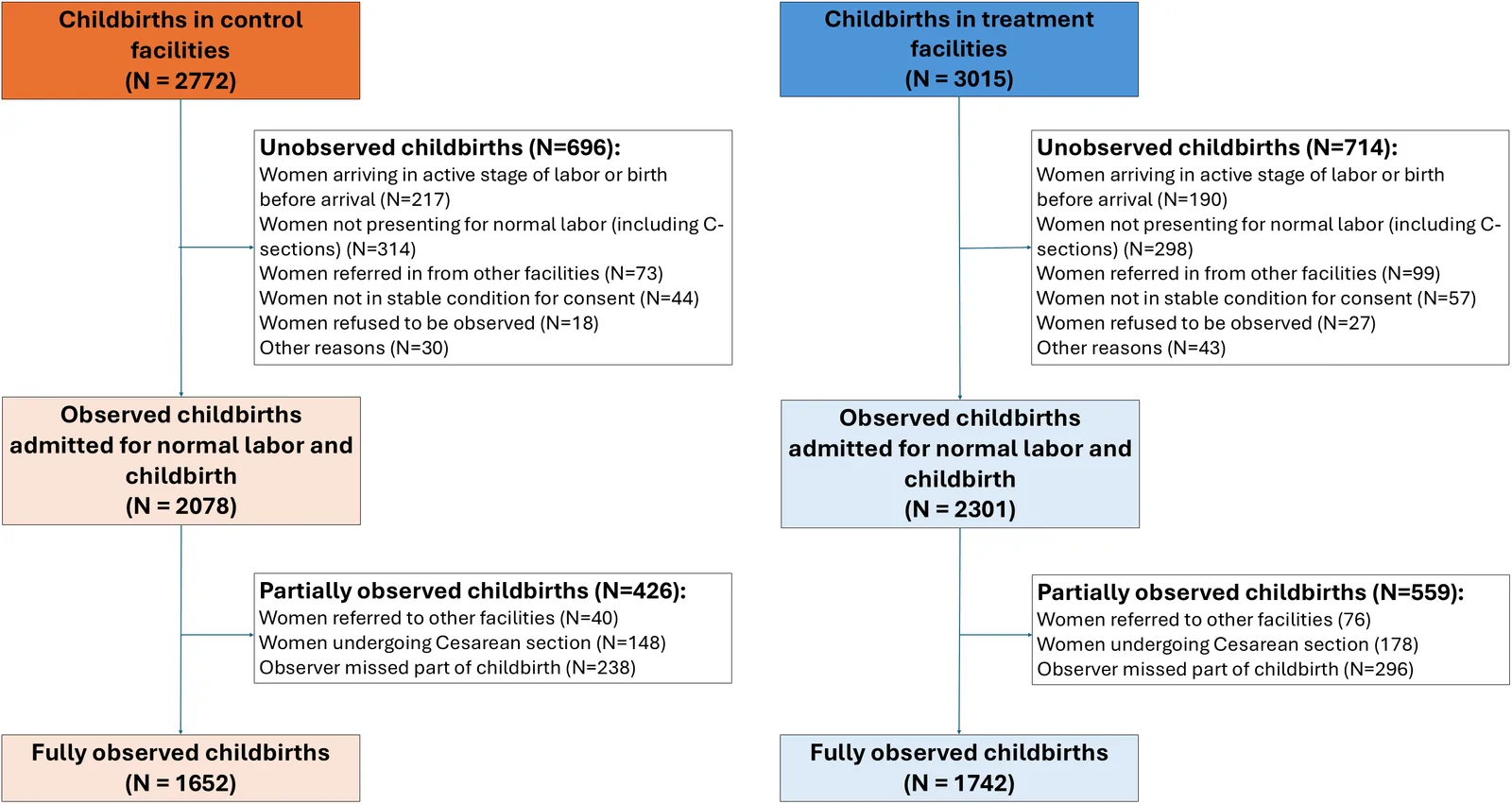
Consort Flow Diagram of Childbirth Observations.

A total of 4,377 admissions were observed for normal labor and childbirth (Fig 1), with 983 observations being incomplete (most commonly because the observation began after the first exam, the patient was referred for cesarean section or transfer upon arrival, or the enumerator’s shift ended before the observation was complete). Patient characteristics from incomplete childbirth observations are reported in Table B in S1 Appendix. Among the remaining fully observed births, patient characteristics were similar between the intervention and control groups (Table 2). For additional clinical context, Table C in S1 Appendix reports rates of maternal and neonatal complications and adverse outcomes observed during childbirth; these outcomes may be influenced by care received during labor and delivery and are therefore presented separately from the childbirth sample characteristics.

**Table 2:** Patient Characteristics in Childbirth Observations.

Childbirth Sample Characteristic^1^	Control (N = 1652)	Intervention (N = 1742)
Age, mean (SD)	25.70 (6.02)	25.41 (5.90)
Married, n (%)	1383 (85.5%)	1429 (88.5%)
Parity, mean (SD)	1.46 (1.56)	1.33 (1.46)
Gravidae, mean (SD)	2.54 (1.64)	2.42 (1.53)
# of antenatal care visits, mean (SD)	3.71 (1.39)	3.58 (1.46)
# of babies delivered, mean (SD)	1.01 (0.09)	1.02 (0.16)
Birthweight in grams, mean (SD)	3078.91 (474.63)	3026.12 (490.44)

Notes:

1. Sample characteristics were extracted from facility maternity registers by enumerators during childbirth observations.

The average age of mothers giving birth in control facilities was 25.7 compared to 25.4 among intervention facilities. Pregnant women also attended a similar number of antenatal care visits, with an average of 3.7 visits for women observed in control facilities and 3.6 in intervention facilities. The average documented birthweight for observed births was 3,078.9 grams in control facilities and 3,026.1 grams in intervention facilities.

**Fig 2:**
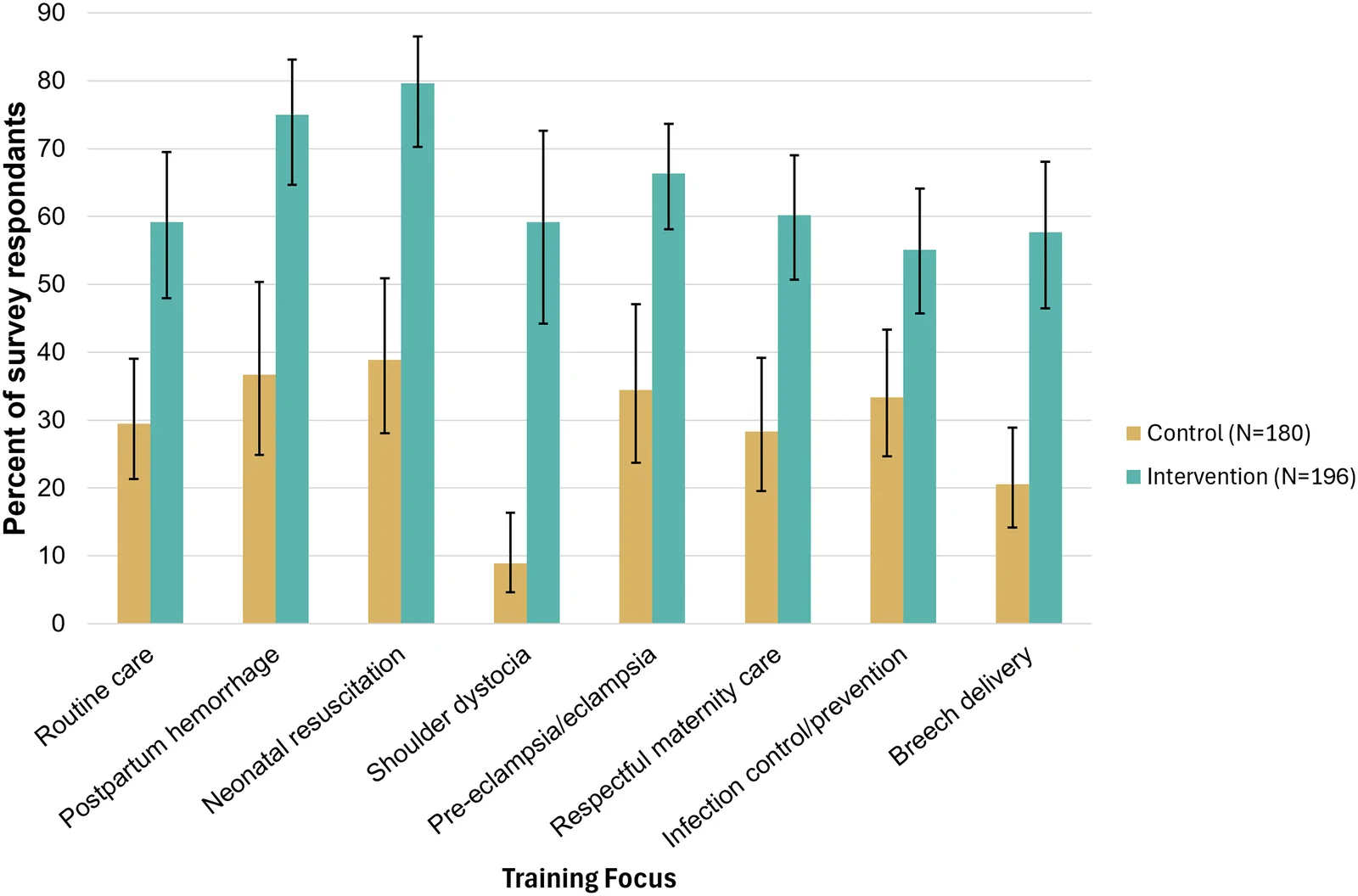
In-service trainings attended by providers in the last 6 months (endline survey).

Receipt of training varied substantially across study facilities. The number of CME sessions and simulation drills conducted over the study period ranged from 5 to 19 and 8–17, respectively. At endline, provider interviews indicated that a higher proportion of providers in intervention facilities attended in-service trainings, across all eight types assessed, compared to control facilities (Fig 2). These included trainings conducted by Jacaranda Health and in-service trainings offered as part of providers’ routine workflow. For routine care in-service trainings, 59% of providers in intervention facilities compared to 29% of providers in control facilities reported attending a training in the last 6 months.

Timeliness of care outcomes were not analyzed due to substantial measurement error in recorded time stamps, with 25% of observations having missing or implausible values.

### Provider knowledge

**Fig 3:**
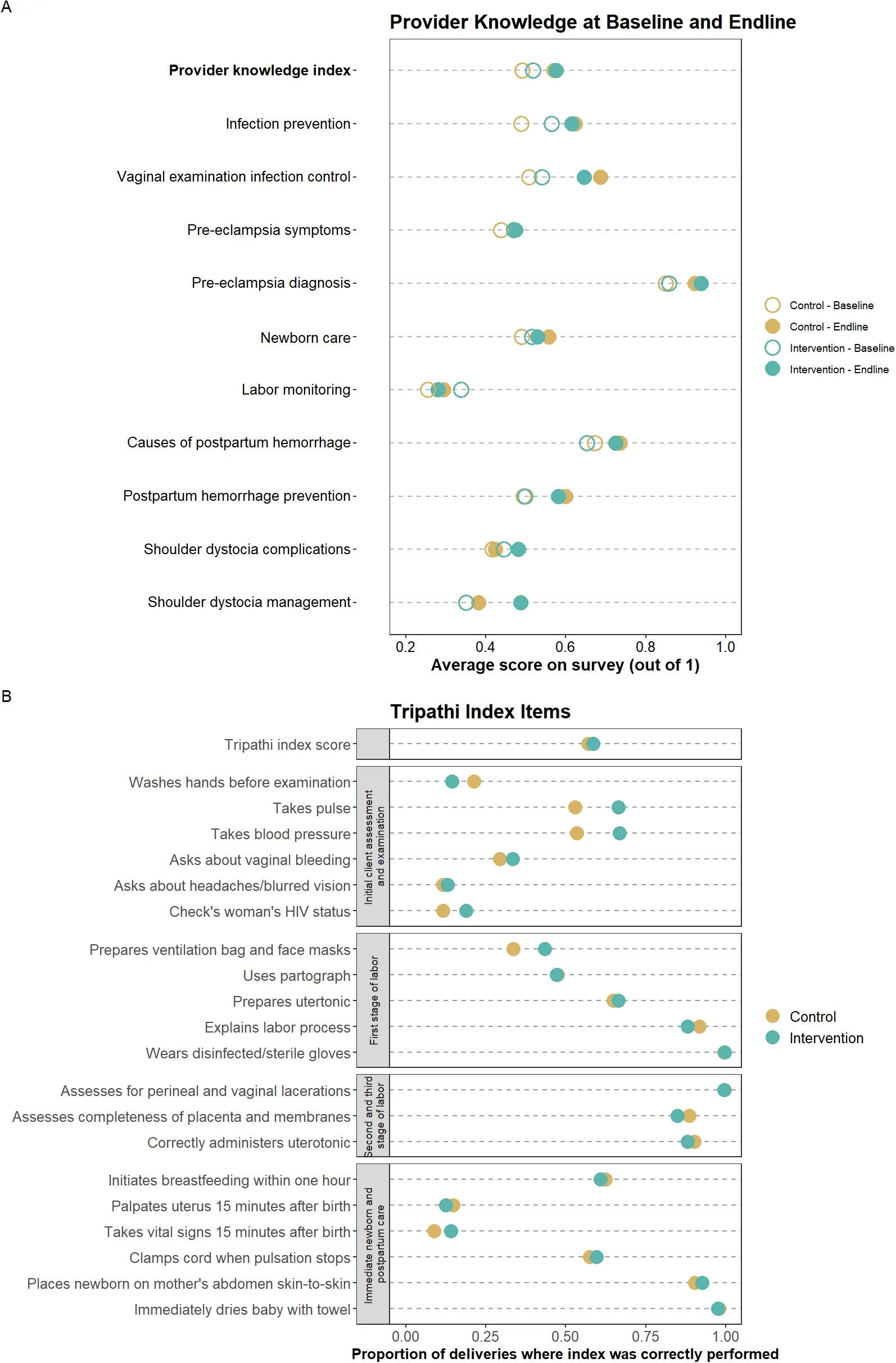
Provider knowledge and performance of essential routine care items during childbirth in intervention and control facilities.

At baseline, the average provider knowledge index was 0.52 (out of 1) in intervention facilities, and 0.49 in control facilities, indicating that providers correctly responded to half of questions on a knowledge assessment of maternal and newborn health topics. By endline, provider knowledge scores improved to 0.58 in the intervention group and 0.57 in the control group, with no statistically significant differences (95% CI: -0.038, 0.051; P = 0.772) between groups (Table 3). Fig 3A details provider scores across each of the 10 knowledge topics. Providers scored lowest on labor monitoring, with only 28% in intervention facilities and 29% in control facilities correctly specifying monitoring frequency for vital signs and vaginal bleeding during the fourth stage of labor. Scores were highest on pre-eclampsia diagnosis, with 94% of providers in the intervention group and 92% in the control group correctly diagnosing pre-eclampsia.

**Table 3:** Intervention Impact on Primary and Secondary Outcomes at Endline.

	Control Mean	Intervention Mean	Unadjusted Effect (95% CI)
***Primary Outcomes***			
Provider Knowledge Index^1^	0.570	0.576	0.006
			(-0.038, 0.051)
			P = 0.772
20-Item Tripathi Index: Quality of Routine Maternity Care	0.570	0.586	0.018
			(-0.029, 0.065)
			P = 0.446
*Tripathi index stages (exploratory outcomes):*			
Initial client assessment and examination	0.399	0.447	0.044
			(-0.040, 0.128)
			P = 0.296
First stage of labor	0.594	0.612	0.023
			(-0.068, 0.114)
			P = 0.616
Second and third stage of labor	0.930	0.910	-0.020
			(-0.056, 0.016)
			P = 0.267
Immediate newborn and postpartum care	0.553	0.563	0.004
			(-0.004, 0.013)
			P = 0.297
Quality of care for management of neonatal resuscitation in simulation^2^	0.700	0.791	0.094*
			(0.00, 0.188)
			P = 0.050
Full score on neonatal resuscitation simulation^2^ (exploratory outcome)	0.039	0.209	0.172**
			(0.081, 0.263)
			P < 0.001
***Secondary Outcomes***			
Provider motivation and satisfaction^3^	0.793	0.809	0.016
			(-0.017, 0.050)
			P = 0.326
Quality of care for management of maternal and neonatal complications^4^	0.847	0.855	0.012
			(-0.046, 0.069)
			P = 0.687
Quality of care for management of neonatal resuscitation (exploratory outcome)	0.790	0.815	0.041
			(-0.001, 0.083)
			P = 0.056
Successful neonatal resuscitation (exploratory outcome)	0.903	0.959	0.062
			(-0.009, 0.133)
			P = 0.088
Quality of care for management of postpartum hemorrhage (exploratory outcome)	0.955	0.917	-0.039
			(-0.092, 0.013)
			P = 0.141
Postpartum bleeding stabilized (exploratory outcome)	0.982	0.925	-0.076
			(-0.180, 0.027)
			P = 0.147
Quality of care for infection prevention^5^	0.797	0.766	-0.030
			(-0.077, 0.017)
			P = 0.203
Partograph completion^6^	0.686	0.682	-0.002
			(-0.102, 0.098)
			P = 0.973
Interpersonal abuse^7^	0.121	0.102	-0.025
			(-0.076, 0.026)
			P = 0.333
Unsupportive birth environment (0–12)^8^	3.348	3.581	0.245
			(-0.371, 0.861)
			P = 0.426
Severe maternal and neonatal morbidity and mortality^9^	0.102	0.103	-0.008
			(-0.036, 0.020)
			P = 0.545

** p < 0 ∙ 01, * p < 0 ∙ 05

Notes:

1. Provider knowledge was assessed based on 10 multiple response/open-ended questions in the provider survey, with each question being scored out of 1. The provider knowledge index is calculated as the fraction of correct responses to the 10 knowledge questions.

2. Quality of care for neonatal resuscitation was measured as the proportion of the following action that were performed during the simulation: checked equipment and selected correctly sized mask; performed initial steps in stabilization; checked baby’s airway, breathing pattern, and pulse; shouted for help; applied mask to make a firm seal; started ventilating; ventilated 40–60 breaths in the first minute; chest rose during ventilation breaths; repositioned mask/baby rechecked the baby’s breathing and pulse after first minute of ventilation, correctly responded to hypothetical scenario 1, correctly responded to hypothetical scenario 2, gave correct instructions to mother following resuscitation.

3. Provider motivation, scored from 0-1, was calculated based on 11 Likert scale questions asking about job motivation and satisfaction. Responses for each question (on a 1–5 scale, with 1 denoting “strongly disagree” and 5 denoting “strongly agree”) were summed and divided by 55. A higher score corresponds to higher self-rated motivation.

4. Measured as the fraction of essential actions that providers ought to have performed in the management of relevant maternal and neonatal complications, including neonatal resuscitation, postpartum hemorrhage, shoulder dystocia, and pre-eclampsia/eclampsia.

5. Infection prevention measures assessed include handwashing with soap and water or use of an alcohol-based hand rub before every exam; use of high-level disinfected or sterile gloves for every vaginal exam; immediate disposal of sharps in a puncture-proof container; sterilization or high-level disinfection of reusable instruments; disposal of contaminated waste in leak-proof containers; and timely cleanup of blood and body fluid spills.

6. Partograph completion was measured as the fraction of 10 steps performed to plot labor progress appropriately and comprehensively on a patient’s partograph.

7. Interpersonal abuse is a binary indicator measuring whether the mother was shouted/screamed at, insulted, scolded, mocked, threatened, physically abused, or received negative comments at any point during the childbirth observation.

8. Unsupportive birth environment was based on a 12 item index, where a higher score corresponds to a more unsupportive birth environment.

9. A binary indicator for postpartum hemorrhage, severe pre-eclampsia, eclampsia, maternal sepsis, neonatal sepsis or systemic infection, uterine rupture, neonatal asphyxia, maternal mortality, and perinatal mortality.

### Quality of routine and emergency obstetric and newborn care

Based on the 20-item quality of care index by Tripathi et al., providers in intervention facilities correctly performed 58.6% of the assessed items, compared to 57.0% in control facilities, with an observed intra-cluster correlation of 0.36 (Fig 3B). Essential actions were least likely to be completed during initial client assessment and examination. Providers in intervention facilities asked mothers about their HIV status in only 18.8% (11.7% in control facilities) of births, inquired about headaches or blurred vision in 13.1% (11.7%) of births, and washed hands or used alcohol rubs in just 14.5% (21.2%) of births. The highest rates of adherence were in the second and third stages of labor where providers in intervention facilities correctly performed 91.0% of essential items (93.0% in control facilities). In the immediate newborn and postpartum period, palpation of the uterus 15 minutes after birth occurred in only 12.6% of births in intervention facilities and 14.8% in control facilities, while checking vital signs at this interval occurred in just 14.2% and 8.8%, respectively. At endline, there were no significant differences in the overall quality of routine maternity care between intervention and control groups (95% CI: -0.029, 0.065; P = 0.446; Table 3).

### Quality of care in neonatal resuscitation simulation

Providers in intervention facilities scored higher on the neonatal resuscitation simulation, correctly performing 79.1% of simulation steps compared to 70.0% of simulation steps in control facilities (95% CI: 0.000, 0.188; P = 0.050; Table 3). In exploratory analyses, we found providers in the intervention group were 17.2 percentage points (95% CI: 8.1, 26.3; P < 0.001; Table 3) more likely to achieve a perfect score on the simulation compared to a control group rate of 3.9%.

### Secondary outcomes

Analysis of pre-registered secondary outcomes showed no significant improvement in provider motivation and satisfaction, management of maternal and neonatal complications (in aggregate), quality of infection prevention, quality of partograph completion, and patient treatment during labor and childbirth (Table 3). In exploratory analyses of standardized childbirth observations, we found no statistically significant improvements in neonatal resuscitation quality and successful resuscitation, as well as postpartum hemorrhage management. However, the estimated effects for neonatal resuscitation quality (95% CI: -0.001, 0.083; P = 0.056) and successful resuscitation (95% CI: -0.009, 0.013; P = 0.088) were positive but did not meet the pre-specified 5% significance threshold.

### Sensitivity analysis

In adjusted analyses, the improvement in the primary simulation-based neonatal resuscitation outcome was only marginally significant (Table D in S1 Appendix); after applying Benjamini-Hochberg adjustments for multiple comparisons and excluding the three facilities exposed to other training interventions, the improvement was no longer statistically significant (Tables E and F in S1 Appendix). In contrast, improvements in the proportion of providers achieving a perfect score on the resuscitation simulation remained significant across sensitivity analyses (Tables D and F in S1 Appendix).

## Discussion

We conducted a cluster RCT to evaluate the impact of an onsite nurse mentorship and simulation training intervention on provider knowledge, quality of childbirth care, and maternal and neonatal health outcomes in Kenya. The bundled mentorship intervention included monthly CME lectures and simulation drills focused on basic and emergency obstetric and newborn care over a period of nine months. While the intervention increased the likelihood that providers had received training, we did not observe improvements in provider knowledge or care metrics such as quality of routine maternity care, partograph completion, infection prevention, and management of neonatal and maternal complications in aggregate. However, providers in intervention facilities demonstrated higher-quality neonatal resuscitation care in simulation-based assessments, with suggestive evidence of concordant improvements during standardized childbirth observations. Although these specific findings were not robust across all sensitivity analyses, improvements in the proportion of providers achieving a perfect simulation score were robust at the 5% level. These findings are particularly relevant given that birth asphyxia is among the most common obstetric emergencies in Kenya and a leading cause of neonatal mortality. In contrast, we did not observe improvements in postpartum hemorrhage management, which, unlike neonatal resuscitation, was not separately evaluated through a standardized simulation assessment at endline.

These findings contribute to a mixed literature on the effectiveness of provider training interventions in low-resource settings. While several studies have reported positive impacts of training programs on provider knowledge and performance, many rely on pre-post designs without control groups, limiting their ability to attribute observed changes to the intervention itself [10,30]. Among randomized trials, results have been inconsistent. One randomized evaluation in Mexico found improvements in selected clinical practices following simulation-based training, though these gains were limited to targeted behaviors rather than broader measures of care quality [31]. A large cluster RCT in Kenya and Uganda reported reductions in neonatal mortality, specifically among preterm and low-birthweight newborns, when simulation-based training was embedded within a broader quality improvement package [32]. In contrast, an RCT conducted in Tanzania by Larson et al. (2020) found limited impacts of in-service training combined with mentorship and infrastructure support on quality improvement [33]. A separate trial of a coaching-based implementation of the WHO Safe Childbirth Checklist improved adherence to essential practices but did not translate into reductions in maternal or perinatal mortality [19].

The limited impact of the MENTORS intervention may reflect aspects of how the training was delivered rather than, or in addition to, its overall frequency. While facilities conducted regular CME lectures and simulation drills over the nine-month period, these sessions covered a broad range of topics, which may have limited opportunities for repeated practice and reinforcement of specific skills. Prior studies demonstrating positive impacts from mentorship interventions often involved a low-dose high-frequency training model with providers receiving several consecutive days of training supplemented by refresher sessions [17,34,35]. Additionally, although a higher proportion of providers in intervention facilities reported receiving in-service training on routine childbirth care compared to controls (59% vs. 29%), a substantial proportion of providers in the intervention arm still had not received recent training, potentially diluting any impact of the intervention.

Implementation challenges likely further constrained impact. During the study period, at least six mentor turnovers were reported across participating facilities, although the true number may have been higher. Providers may have also crossed over between intervention and control facilities during the study period, reducing differences between the study groups. Facilities also experienced staffing shortages that disrupted scheduling and training. It is possible that challenges such as limited staffing, competing responsibilities, and lack of protected time made it difficult for providers to attend training sessions, suggesting a potential mismatch between the intervention design and day-to-day provider workflows. Additionally, variability in baseline facility infrastructure and resource ability may have limited providers’ ability to implement new skills and/or maintain improvements in care quality [36]. These factors likely reduced both the fidelity and the intensity of implementation. At the same time, improvements in provider knowledge and care practices observed in both intervention and control facilities across the study period raise the possibility that external or concurrent quality improvement efforts we were unaware of may have influenced outcomes across both arms.

Finally, the limited impact of the intervention on our primary outcomes may reflect variation in implementation of the MENTORS curriculum across facilities. Although the program addressed both routine and emergency obstetric and newborn care, in-facility mentors tailored training to local needs, and routine care may not have been emphasized uniformly across sites. Nevertheless, we selected routine maternity care quality as a primary outcome because these practices affect a large share of patients, offering greater statistical power to detect meaningful changes. By contrast, obstetric emergencies, while clinically critical, occur relatively infrequently, limiting power to detect differences in such outcomes. Moreover, improving the quality of routine care may offer broader opportunities to prevent complications and avertable maternal and neonatal deaths.

Still, the intervention improved performance on the broadly administered neonatal resuscitation simulation, the sole primary outcome focused on emergency care, with exploratory analyses suggesting concordant improvements in childbirth observations. More widespread incorporation of simulations or drills targeting routine care practices – as well as secondary outcomes like partograph completion, infection prevention, and respectful maternity care – may likewise have produced stronger effects on those outcomes. This aligns with earlier work showing that frequent, focused drills can lead to sustained improvements in emergency preparedness and neonatal outcomes [10,17].

Key strengths of this study include its randomized design and the broad geographic coverage of study facilities, which included 40 facilities across eight counties in Kenya. We assessed the impact of the MENTORS intervention across multiple levels, including provider knowledge and skills, changes in clinical practice, and improvements in maternal and neonatal health outcomes. A particularly notable strength was the large volume of childbirth observations collected by trained enumerators at each of these study facilities using standardized protocols.

Nevertheless, there are several limitations to our study. To the extent that women gave birth in facilities in which they sought antenatal care, births observed in intervention facilities were likely of women who were also exposed to the PROMPTS digital health platform. While births were balanced on measured patient characteristics, PROMPTS may have introduced unmeasured differences in maternal knowledge, preparedness, or engagement with care that could have influenced observed delivery care quality. Our findings are also limited in generalizability, as our study was restricted to mid- to high-volume facilities (50–400 births per month); thus, the generalizability of our findings to lower-volume or more remote facilities remains uncertain. We also acknowledge that unmeasured post-randomization differences between facilities that were not directly related to the intervention cannot be fully characterized and may have contributed to outcome differences. In addition, the study was not sufficiently powered to detect effects on rarer emergency obstetric outcomes, such as postpartum hemorrhage and eclampsia, which were areas emphasized in the program’s content. While Jacaranda Health tracked mentorship activities held at the facility level, data on individual provider attendance were not collected. Thus, we could not link training participation to the providers observed at endline and are unable to comment on the extent to which individual providers were directly exposed to the intervention. Additionally, the types of trainings delivered varied across facilities, as facility mentors selected trainings based on their assessed need, which further introduced variation in exposure to the intervention. While the improvement in the primary simulation-based neonatal resuscitation outcome was not robust across all sensitivity analyses, the direction and magnitude of the effect – as well as concordant exploratory results in childbirth observations – suggest a meaningful difference between intervention and control facilities. Nevertheless, because providers in intervention facilities had greater exposure to simulation-based training, improved performance may partly reflect increased familiarity with the simulation format and assessment environment, in addition to gains in clinical skill. Finally, while our primary measure of childbirth quality was based on direct childbirth observations, which allowed us to directly capture provider actions rather than rely on self-reports or facility records, they may have been subject to the Hawthorne effect, wherein providers altered their behavior due to being observed. We consider this risk to be minimal, as overall quality remained low across both groups and provider scores were consistent between earlier and later observation days.

To conclude, despite modest increases in training exposure and the quality of neonatal resuscitation care, we did not identify improvements across other broad measures of provider knowledge or routine and emergency childbirth care quality. Our results suggest that insufficient intervention intensity, limited workflow integration, and/or lack of ongoing reinforcement may prevent mentorship programs from achieving their intended impact. Future research should explore how mentorship interventions can be optimized for greater effectiveness, whether through increased frequency and duration, integration with existing supervision systems, more focus on simulation-based training, and/or better alignment with provider incentives and facility capacity. Further work is needed to understand the specific mechanisms through which mentorship affects provider behavior and to identify which domains of care are most amenable to change. In addition, future research should examine the role of implementation fidelity, including mentor quality and the consistency and quality of training sessions, in shaping program effectiveness.

## Supporting information

S1 Appendix Fig A. Map of study facilities. Fig B. MENTORS training-of-trainers program structure. Fig C. Timeline of study activities. Table A. Index components for primary and secondary outcomes. Table B. Patient characteristics among incomplete childbirth observations. Table C. Frequency of childbirth complications among completed childbirth observations. Table D. Adjusted intervention impact on primary and secondary outcomes. Table E. Intervention impact on pre-registered outcomes with Benjamini–Hochberg adjusted P-values. Table F. Intervention impact on outcomes excluding three facilities with ongoing similar interventions.
(DOCX)

S1 Checklist CONSORT 2025. (DOCX)
