# Supplementary material for: Impact of a long-term mentorship program for childbirth providers on quality of care: A cluster randomized controlled trial
Source: PLOS Glob Public Health. 2026 Sep 2;6(9):e0006073. doi: 10.1371/journal.pgph.0006073 (PMC13537552; doi:10.1371/journal.pgph.0006073)
Supplement: Supplementary file 1 [file pgph.0006073.s001.docx]

**S1 Appendix**

**Fig A: Map of study facilities**

Note: Map data courtesy of <https://open.africa/dataset/kenya-counties-shapefile/resource/0b78f25e-494e-4258-96b8-a3ab2b35b121>; available under a Creative Commons Attribution license.

**Fig B: MENTORS training of trainers program structure**

**Fig C: Timeline of study activities**

**Table A: Index components for primary and secondary outcomes**

**Outcome Measures**

Quality and Timeliness of Routine Maternity Care (20-item Tripathi Index)

Initial client assessment and examination

Provider asked about mother’s HIV status and/or offered HIV test

Provider asked if mother experienced headaches or blurred vision

Provider asked if mother experienced vaginal bleeding

Provider took mother’s blood pressure

Provider took mother’s pulse

Provider washed hands or used alcohol rub before initial exam

First stage of labor

Provider wore disinfected or sterile gloves for any vaginal exam

Provider explained what will happen in labor to mother/support person

Provider prepared uterotonic drug to use for active management of the third stage of labor

Provider used partograph to monitor progress of labor

Provider was prepared with ventilation bag + newborn face masks

Second and third stage of labor

Provider correctly administered uterotonic (timing, dose, route)

Provider assessed completeness of placenta and membranes

Provider assessed for perineal and vaginal lacerations

Immediate newborn and postpartum care

Provider immediately dried baby with towel

Provider immediately placed baby on mother for skin-to-skin

Provider tied/clamped cord in sterile fashion with adequate delay

Provider checked mother’s HR and BP within 15 minutes of birth

Provider palpated uterus (for firmness) within 15 minutes of birth

Provider assisted mother to start breastfeeding within 1 hour of birth

Provider Knowledge on Obstetric and Newborn Care

Universal precautions for infection prevention

Proper infection control before a vaginal examination

Symptoms of pre-eclampsia

Immediate newborn care and health checks after childbirth

Frequency of monitoring for vital signs and vaginal bleeding in the 4th stage of labor

Primary causes of postpartum hemorrhage

Prevention of postpartum hemorrhage

Maternal complications of shoulder dystocia

Management of shoulder dystocia

Diagnosing pre-eclampsia

Quality of Care for Management of Neonatal Resuscitation (Simulation)

Provider checked equipment (tested function) and selected correctly sized mask

Provider performed initial steps in stabilization (dry/stimulate baby)

Provider checked baby’s airway, breathing pattern, and pulse

Provider shouted for help

Provider applied mask to make a firm seal

Provider started ventilating

Provider planned to ventilate correct # of breaths (40-60) in first minute

Chest rose during ventilation breaths

Provider repositioned mask/baby if no chest rise

Provider rechecked the baby’s breathing and pulse after first minute of ventilation

Provider gave compressions and ventilations in a 3:1 ratio when told that baby not independently breathing and has a heart rate of 40

Provider said they would give oxygen if baby breathing independently but with difficulty and with heart rate of 100

Provider instructed mother to continue monitoring baby’s breathing following successful resuscitation

Provider Motivation and Satisfaction (all components scored on Likert scale from strongly disagree [1] to strongly agree [5])

I have no motivation at work

Overall, I am very satisfied with my job

I am satisfied with the opportunity to use my abilities in my job

My job makes me feel good about myself

I am very proud to be working for this facility

I am glad that I work for this facility rather than other facilities in the country

This facility really inspires me to do my very best on the job

I always complete my tasks efficiently and correctly

I am a hard worker

I am punctual about coming to work

I am confident that I could manage obstetric emergencies well

Quality of Care for Newborn Resuscitation (each component only assessed if clinically indicated)

Provider stimulated baby with back rubbing

Provider appropriately called for help

Help arrived for provider

Provider tied or clamped cord immediately

Provider cut cord with sterile blade/scissor

Provider placed newborn on back on clean, warm surface or towel

Provider placed newborn’s head in slightly extended position to open airway

Providers told mother/support person what was going to be done

Provider listened to mother and provides support/reassurance

Provider checked newborn’s mouth, back of throat, and nose or secretions

Provider placed mask on newborn’s face so it covers chin, mouth, nose (not eyes)

Provider checked seal by ventilating and observing chest rise

Provider readjusted newborn/mask position and repeated attempt at suction and ventilation, as necessary

Newborn’s chest rose in response to ventilation

Provider ventilated at correct rate (40-60 breaths per minute)

Provider conducted assessment of newborn breathing/pulse after 1 minute of ventilation

Provider gave oxygen to newborn

Provider explained to mother/support person what happened

Provider listened to mother and responded attentively to questions

Management of Maternal and Neonatal Complications (each component only assessed if clinically indicated)

Quality of Care for Newborn Resuscitation (see above)

Quality of Care for Management of Postpartum Hemorrhage

Provider monitored bleeding

Provider performed uterine massage

Provider ordered labs

Provider inserted urine catheter

Provider administered IV fluids

Provider administered uterotonic correctly, in terms of dosing

Provider used balloon or condom tamponade

Provider repaired laceration

Provider performed manual removal of placenta

Provider performed hysterectomy

Provider gave blood products

Patient left alone at any point during this section of the observation

Quality of Care for Management of Shoulder Dystocia

Provider called for help

Provider performed any of the following maneuver for shoulder dystocia: episiotomy, McRoberts, suprapubic pressure, internal rotation, removal of posterior arm, rolling patient to hands and knees

Quality of Care for Eclampsia

Provider made plans for expedited childbirth

Provider checked urine protein

Provider ordered preeclampsia labs

Provider started IV fluids

Provider gave magnesium sulfate

Provider gave antihypertensive

Provider checked blood pressure at least once per hour until childbirth

Provider checked respiration at least once per hour until childbirth

Quality of Care for Infection Prevention

Provider always washed hands with soap and water or uses alcohol hand rub before every exam

Provider always wore high-level disinfected or sterile gloves for every vaginal exam

Provider always disposed of sharps in a puncture-proof container right after use

Provider always sterilized or disinfected reusable instruments

Provider always disposed of contaminated waste in leak-proof containers

Provider always cleaned up blood and body fluid spills promptly

Quality of Care for Partograph Completion

Provider used partograph to track labor progress

Provider initiated use of partograph at or > 4 cm dilation

Provider plotted fetal heart rate every ½ hour

Provider recorded color of amniotic fluid at every vaginal exam

Provider plotted cervical dilatation every 4 hours, unless indicated

Provider plotted descent of head every 4 hours

Provider plotted contractions every ½ hour

Provider plotted mother’s pulse every ½ hour

Provider recorded mother’s blood pressure every ½ hour

Provider recorded mother’s temperature every ½ hour

Provider appropriately filled out partograph after childbirth

Interpersonal Abuse

Mother never shouted/screamed at

Mother never insulted/mocked/scolded

Mother never received negative comments about her physical appearance, sexual activity, or her baby’s appearance

Mother was never threatened with physical violence or experienced withheld/inappropriate care

Mother was never physically struck

Mother never experienced forceful restraint or pressure

Unsupportive Birth Environment

Mother not offered pain relief when needed or requested

Interpreter not present when needed

No providers were present when baby was born

Mother not offered labor companion

Mother’s request for medical attention not responded to

Provider asked for a bribe, informal payment, or gift

Mother instructed to clean blood/fluids

Mother did not have easy access to water/fluids

Mother not told she could move around

No curtains/partitions during labor and childbirth

No bed during labor and childbirth

Shared bed during labor and childbirth

**Table B: Patient characteristics among incomplete childbirth observation**

| **Patient Characteristics** | **Control (N = 426)** | **Intervention (N = 559)** |
| --- | --- | --- |
| Age, mean (SD) | 24.70 (6.05) | 25.53 (5.82) |
| Missing, n (%) | 30 (7.0%) | 19 (3.4%) |
| Married, n (%) | 302 (71.2%) | 473 (84.6%) |
| Missing, n (%) | 2 (0.5%) | 0 (0.0%) |
| Parity, mean (SD) | 1.24 (2.14) | 1.04 (1.34) |
| Missing, n (%) | 31 (7.3%) | 17 (3.0%) |
| Gravidae, mean (SD) | 2.26 (1.55) | 2.17 (1.44) |
| Missing, n (%) | 31 (7.3%) | 18 (3.2%) |
| # ANC visits, mean (SD) | 3.85 (1.42) | 4.00 (2.16) |
| Missing, n (%) | 61 (14.3%) | 53 (9.5%) |
| # Babies delivered, mean (SD) | 1.02 (0.14) | 1.01 (0.10) |
| Missing, n (%) | 92 (21.6%) | 166 (29.7%) |
| Birthweight, mean (SD) | 3174 (516) | 3062 (510) |
| Missing, n (%) | 99 (23.2%) | 146 (26.1%) |

Notes:

1. Sample characteristics were extracted from facility Maternity Registers by enumerators during childbirth observations.

**Table C: Frequency of childbirth complications among completed childbirth observations**

| **Childbirth Complications^1^** | **Control (N = 1652)** | **Intervention (N= 1742)** |
| --- | --- | --- |
| Postpartum hemorrhage, n (%) | 54 (3.4%) | 62 (3.6%) |
| Eclampsia/pre-eclampsia, n (%) | 4 (0.2%) | 9 (0.5%) |
| Systemic infection/sepsis, n (%) | 6 (0.4%) | 10 (0.6%) |
| Uterine rupture, n (%) | 0 (0.0%) | 0 (0.0%) |
| Neonatal asphyxia, n (%) | 96 (5.8%) | 83 (4.8%) |
| Maternal mortality, n (%) | 2 (0.1%) | 1 (0.1%) |
| Newborn death, n (%) | 16 (1.0%) | 19 (1.1%) |
| Notes:  1. Sample characteristics were extracted from the patient files and facility maternity registers by enumerators during childbirth observations. | | |

**Table D: Adjusted intervention impact on primary and secondary outcomes**

|  | **Control Mean** | **Adjusted Effect (95% CI)** |
| --- | --- | --- |
| **Primary Outcomes** |  |  |
| Provider Knowledge Index^1^ | 0∙570 | 0∙015 |
|  |  | (-0∙031, 0∙061) |
|  |  | P=0∙527 |
| 20-Item Tripathi Index: Quality of Routine Maternity Care | 0∙570 | 0∙003 |
|  |  | (-0∙023, 0∙030) |
|  |  | P=0∙798 |
| *Tripathi index stages (exploratory outcomes):* |  |  |
| Initial client assessment and examination | 0∙399 | 0∙014 |
|  |  | (-0∙030, 0∙057) |
|  |  | P=0∙528 |
| First stage of labor | 0∙594 | 0∙001 |
|  |  | (-0∙060, 0∙063) |
|  |  | P=0∙963 |
| Second and third stage of labor | 0∙930 | -0∙019 |
|  |  | (-0∙054, 0∙016) |
|  |  | P=0∙284 |
| Immediate newborn and postpartum care | 0∙553 | 0∙005 |
|  |  | (-0∙002, 0∙012) |
|  |  | P=0∙177 |
| Quality of care for management of neonatal resuscitation in simulation^2^ | 0∙700 | 0∙086 |
|  |  | (-0∙005, 0∙177) |
|  |  | P=0∙064 |
| Full score on neonatal resuscitation simulation^2^ (exploratory outcome) | 0∙039 | 0∙141** |
|  |  | (0∙070, 0∙212) |
|  |  | P<0∙001 |
| ***Secondary Outcomes*** |  |  |
| Provider motivation and satisfaction^3^ | 0∙793 | 0∙018 |
|  |  | (-0∙009, 0∙044) |
|  |  | P=0∙183 |
| Quality of care for management of maternal and neonatal complications^4^ | 0∙847 | 0∙017 |
|  |  | (-0∙015, 0∙049) |
|  |  | P=0∙286 |
| Quality of care for management of neonatal resuscitation (exploratory outcome) | 0∙790 | 0∙024 |
|  |  | (-0∙014, 0∙061) |
|  |  | P=0∙217 |
| Successful neonatal resuscitation (exploratory outcome) | 0∙903 | 0∙072 |
|  |  | (-0∙008, 0∙152) |
|  |  | P=0∙076 |
| Quality of care for management of postpartum hemorrhage (exploratory outcome) | 0∙955 | -0∙023 |
|  |  | (-0∙068, 0∙022) |
|  |  | P=0∙314 |
| Postpartum bleeding stabilized (exploratory outcome) | 0∙982 | -0∙043 |
|  |  | (-0∙138, 0∙052) |
|  |  | P=0∙369 |
| Quality of care for infection prevention^5^ | 0∙797 | -0∙009 |
|  |  | (-0∙038, 0∙020) |
|  |  | P=0∙553 |
| Partograph completion^6^ | 0∙686 | -0∙013 |
|  |  | (-0∙098, 0∙072) |
|  |  | P=0∙761 |
| Interpersonal abuse^7^ | 0∙121 | -0∙010 |
|  |  | (-0∙055, 0∙036) |
|  |  | P=0∙673 |
| Unsupportive birth environment (0-12)^8^ | 3∙348 | 0∙291 |
|  |  | (-0∙240, 0∙821) |
|  |  | P=0∙275 |
| Severe maternal and neonatal morbidity and mortality^9^ | 0∙102 | -0∙007 |
|  |  | (-0∙036, 0∙023) |
|  |  | P=0∙654 |

** p<0∙01, * p<0∙05

Notes:

1. Provider knowledge was assessed based on 10 multiple response/open-ended questions in the provider survey, with each question being scored out of 1. The provider knowledge index is calculated as the fraction of correct responses to the 10 knowledge questions.

2. Quality of care for neonatal resuscitation was measured as the proportion of the following action that were performed during the simulation: checked equipment and selected correctly sized mask; performed initial steps in stabilization; checked baby’s airway, breathing pattern, and pulse; shouted for help; applied mask to make a firm seal; started ventilating; ventilated 40-60 breaths in the first minute; chest rose during ventilation breaths; repositioned mask/baby rechecked the baby’s breathing and pulse after first minute of ventilation, correctly responded to hypothetical scenario 1, correctly responded to hypothetical scenario 2, gave correct instructions to mother following resuscitation.

3. Provider motivation, scored from 0-1, was calculated based on 11 Likert scale questions asking about job motivation and satisfaction. Responses for each question (on a 1-5 scale, with 1 denoting “strongly disagree” and 5 denoting “strongly agree”) were summed and divided by 55. A higher score corresponds to higher self-rated motivation.

4. Measured as the fraction of essential actions that providers ought to have performed in the management of relevant maternal and neonatal complications, including neonatal resuscitation, postpartum hemorrhage, shoulder dystocia, and pre-eclampsia/eclampsia.

5. Infection prevention measures assessed include handwashing with soap and water or use of an alcohol-based hand rub before every exam; use of high-level disinfected or sterile gloves for every vaginal exam; immediate disposal of sharps in a puncture-proof container; sterilization or high-level disinfection of reusable instruments; disposal of contaminated waste in leak-proof containers; and timely cleanup of blood and body fluid spills.

6. Partograph completion was measured as the fraction of 10 steps performed to plot labor progress appropriately and comprehensively on a patient’s partograph.

7. Interpersonal abuse is a binary indicator measuring whether the mother was shouted/screamed at, insulted, scolded, mocked, threatened, physically abused, or received negative comments at any point during the childbirth observation.

8. Unsupportive birth environment was based on a 12 item index, where a higher score corresponds to a more unsupportive birth environment.

9. A binary indicator for postpartum hemorrhage, severe pre-eclampsia, eclampsia, maternal sepsis, neonatal sepsis or systemic infection, uterine rupture, neonatal asphyxia, maternal mortality, and perinatal mortality.

**Table E: Intervention impact on pre-registered outcomes: Benjamini – Hochberg adjusted P-values for index components**

|  | **Control Mean** | **Unadjusted Effect (95% CI)** |
| --- | --- | --- |
| ***Primary Outcomes*** | | |
| Provider Knowledge Index^1^ | 0∙570 | 0∙006 |
|  |  | (-0∙038, 0∙051) |
|  |  | P_BH_=0∙858 |
| 20-Item Tripathi Index: Quality of Routine Maternity Care | 0∙570 | 0∙018 |
|  |  | (-0∙029, 0∙065) |
|  |  | P_BH_=0∙743 |
| Quality of care for management of neonatal resuscitation in simulation^2^ | 0∙700 | 0∙094 |
|  |  | (0∙00, 0∙188) |
|  |  | P_BH_=0∙497 |
| ***Secondary Outcomes*** |  |  |
| Provider motivation and satisfaction^3^ | 0∙793 | 0∙016 |
|  |  | (-0∙017, 0∙050) |
|  |  | P_BH_=0∙743 |
| Quality of care for management of maternal and neonatal complications^4^ | 0∙847 | 0∙012 |
|  |  | (-0∙046, 0∙069) |
|  |  | P_BH_=0∙858 |
| Quality of care for infection prevention^5^ | 0∙797 | -0∙030 |
|  |  | (-0∙077, 0∙017) |
|  |  | P_BH_=0∙743 |
| Partograph completion^6^ | 0∙686 | -0∙002 |
|  |  | (-0∙102, 0∙098) |
|  |  | P_BH_=0∙973 |
| Interpersonal abuse^7^ | 0∙121 | -0∙025 |
|  |  | (-0∙076, 0∙026) |
|  |  | P_BH_=0∙743 |
| Unsupportive birth environment (0-12)^8^ | 3∙348 | 0∙245 |
|  |  | (-0∙371, 0∙861) |
|  |  | P_BH_=0∙743 |
| Severe maternal and neonatal morbidity and mortality^9^ | 0∙102 | -0∙008 |
|  |  | (-0∙036, 0∙020) |
|  |  | P_BH_=0∙778 |

** p<0∙01, * p<0∙05

P_BH_: Benjamini Hochberg adjusted P-values.

Notes:

1. Provider knowledge was assessed based on 10 multiple response/open-ended questions in the provider survey, with each question being scored out of 1. The provider knowledge index is calculated as the fraction of correct responses to the 10 knowledge questions.

2. Quality of care for neonatal resuscitation was measured as the proportion of the following action that were performed during the simulation: checked equipment and selected correctly sized mask; performed initial steps in stabilization; checked baby’s airway, breathing pattern, and pulse; shouted for help; applied mask to make a firm seal; started ventilating; ventilated 40-60 breaths in the first minute; chest rose during ventilation breaths; repositioned mask/baby rechecked the baby’s breathing and pulse after first minute of ventilation, correctly responded to hypothetical scenario 1, correctly responded to hypothetical scenario 2, gave correct instructions to mother following resuscitation.

3. Provider motivation, scored from 0-1, was calculated based on 11 Likert scale questions asking about job motivation and satisfaction. Responses for each question (on a 1-5 scale, with 1 denoting “strongly disagree” and 5 denoting “strongly agree”) were summed and divided by 55. A higher score corresponds to higher self-rated motivation.

4. Measured as the fraction of essential actions that providers ought to have performed in the management of relevant maternal and neonatal complications, including neonatal resuscitation, postpartum hemorrhage, shoulder dystocia, and pre-eclampsia/eclampsia.

5. Infection prevention measures assessed include handwashing with soap and water or use of an alcohol-based hand rub before every exam; use of high-level disinfected or sterile gloves for every vaginal exam; immediate disposal of sharps in a puncture-proof container; sterilization or high-level disinfection of reusable instruments; disposal of contaminated waste in leak-proof containers; and timely cleanup of blood and body fluid spills.

6. Partograph completion was measured as the fraction of 10 steps performed to plot labor progress appropriately and comprehensively on a patient’s partograph.

7. Interpersonal abuse is a binary indicator measuring whether the mother was shouted/screamed at, insulted, scolded, mocked, threatened, physically abused, or received negative comments at any point during the childbirth observation.

8. Unsupportive birth environment was based on a 12 item index, where a higher score corresponds to a more unsupportive birth environment.

9. A binary indicator for postpartum hemorrhage, severe pre-eclampsia, eclampsia, maternal sepsis, neonatal sepsis or systemic infection, uterine rupture, neonatal asphyxia, maternal mortality, and perinatal mortality.

**Table F: Intervention impact on primary and secondary outcomes excluding facilities (n=3) with ongoing similar interventions**

|  | **Control Mean** | **Unadjusted Effect (95% CI)** |
| --- | --- | --- |
| ***Primary Outcomes*** | | |
| Provider Knowledge Index^1^ | 0∙570 | -0∙008 |
|  |  | (-0∙048, 0∙032) |
|  |  | P=0∙699 |
| 20-Item Tripathi Index: Quality of Routine Maternity Care | 0∙570 | 0∙016 |
|  |  | (-0∙031, 0∙063) |
|  |  | P=0∙504 |
| Quality of care for management of neonatal resuscitation in simulation^2^ | 0∙700 | 0∙061 |
|  |  | (-0∙036, 0∙158) |
|  |  | P=0∙211 |
| Full score on neonatal resuscitation simulation^2^ (exploratory outcome) | 0∙039 | 0∙131** |
|  |  | (0∙050, 0∙213) |
|  |  | P=0∙002 |
| ***Secondary Outcomes*** |  |  |
| Provider motivation and satisfaction^3^ | 0∙793 | 0∙004 |
|  |  | (-0∙033, 0∙041) |
|  |  | P=0∙830 |
| Quality of care for management of maternal and neonatal complications^4^ | 0∙847 | -0∙002 |
|  |  | (-0∙056, 0∙053) |
|  |  | P=0∙954 |
| Quality of care for management of neonatal resuscitation (exploratory outcome) | 0∙790 | 0∙032 |
|  |  | (-0∙012, 0∙076) |
|  |  | P=0∙147 |
| Successful neonatal resuscitation (exploratory outcome) | 0∙903 | 0∙080* |
|  |  | (0∙019, 0∙141) |
|  |  | P=0.010 |
| Quality of care for management of postpartum hemorrhage (exploratory outcome) | 0∙955 | -0∙055* |
|  |  | (-0∙108, -0∙001) |
|  |  | P=0∙046 |
| Postpartum bleeding stabilized (exploratory outcome) | 0∙982 | -0∙062 |
|  |  | (-0∙161, 0∙038) |
|  |  | P=0∙222 |
| Quality of care for infection prevention^5^ | 0∙797 | -0∙037 |
|  |  | (-0∙091, 0∙016) |
|  |  | P=0∙166 |
| Partograph completion^6^ | 0∙686 | 0∙028 |
|  |  | (-0∙074, 0∙129) |
|  |  | P=0∙584 |
| Interpersonal abuse^7^ | 0∙121 | -0∙021 |
|  |  | (-0∙078, 0∙036) |
|  |  | P=0∙455 |
| Unsupportive birth environment (0-12)^8^ | 3∙348 | 0∙200 |
|  |  | (-0∙455, 0∙854) |
|  |  | P=0∙540 |
| Severe maternal and neonatal morbidity and mortality^9^ | 0∙102 | -0∙005 |
|  |  | (-0∙031, 0∙020) |
|  |  | P=0∙677 |

** p<0∙01, * p<0∙05

Notes:

1. Provider knowledge was assessed based on 10 multiple response/open-ended questions in the provider survey, with each question being scored out of 1. The provider knowledge index is calculated as the fraction of correct responses to the 10 knowledge questions.

2. Quality of care for neonatal resuscitation was measured as the proportion of the following action that were performed during the simulation: checked equipment and selected correctly sized mask; performed initial steps in stabilization; checked baby’s airway, breathing pattern, and pulse; shouted for help; applied mask to make a firm seal; started ventilating; ventilated 40-60 breaths in the first minute; chest rose during ventilation breaths; repositioned mask/baby rechecked the baby’s breathing and pulse after first minute of ventilation, correctly responded to hypothetical scenario 1, correctly responded to hypothetical scenario 2, gave correct instructions to mother following resuscitation.

3. Provider motivation, scored from 0-1, was calculated based on 11 Likert scale questions asking about job motivation and satisfaction. Responses for each question (on a 1-5 scale, with 1 denoting “strongly disagree” and 5 denoting “strongly agree”) were summed and divided by 55. A higher score corresponds to higher self-rated motivation.

4. Measured as the fraction of essential actions that providers ought to have performed in the management of relevant maternal and neonatal complications, including neonatal resuscitation, postpartum hemorrhage, shoulder dystocia, and pre-eclampsia/eclampsia.

5. Infection prevention measures assessed include handwashing with soap and water or use of an alcohol-based hand rub before every exam; use of high-level disinfected or sterile gloves for every vaginal exam; immediate disposal of sharps in a puncture-proof container; sterilization or high-level disinfection of reusable instruments; disposal of contaminated waste in leak-proof containers; and timely cleanup of blood and body fluid spills.

6. Partograph completion was measured as the fraction of 10 steps performed to plot labor progress appropriately and comprehensively on a patient’s partograph.

7. Interpersonal abuse is a binary indicator measuring whether the mother was shouted/screamed at, insulted, scolded, mocked, threatened, physically abused, or received negative comments at any point during the childbirth observation.

8. Unsupportive birth environment was based on a 12 item index, where a higher score corresponds to a more unsupportive birth environment.

9. A binary indicator for postpartum hemorrhage, severe pre-eclampsia, eclampsia, maternal sepsis, neonatal sepsis or systemic infection, uterine rupture, neonatal asphyxia, maternal mortality, and perinatal mortality.
